# Crystal structure of (*E*)-4-benzyl­idene-6-phenyl-1,2,3,4,7,8,9,10-octa­hydro­phenanthridine

**DOI:** 10.1107/S2056989017009537

**Published:** 2017-06-30

**Authors:** Baidaa K. Al-Rubaye, Alice Brink, Gary J. Miller, Herman Potgieter, Mohamad J. Al-Jeboori

**Affiliations:** a Department of Chemistry, College of Education for Pure Science (Ibn Al-Haitham), University of Baghdad, Iraq; b Department of Chemistry, University of the Free State, PO Box 339, Bloemfontein, South Africa; c Analytical Sciences, Manchester Metropolitan University, Chester Street M1 5GD, UK; d School of Research, Enterprise & Innovation, Manchester Metropolitan University, Chester Street, Manchester M1 5GD, UK; e School of Chemical & Metallurgical Engineering, University of the Witwatersr, Private Bag X3, Wits 2050, South Africa

**Keywords:** crystal structure, Mannich reaction, phenanthridine moiety, C—H⋯N inter­actions, π–π inter­actions, Hirschfeld surfaces

## Abstract

The preparation of the title compound, C_26_H_25_N, was achieved by the condensation of an ethano­lic mixture of benzaldehyde, cyclo­hexa­none and ammonium acetate in a 2:1:1 molar ratio. There are two crystallographically independent mol­ecules in the asymmetric unit. The two cyclo­hexyl rings adopt an *anti*-envelope conformation with the benzyl moiety adopting a *cis* conformation with respect to the nitro­gen atom of the phenanthridine segment. In the crystal, mol­ecules are linked through C—H⋯N inter­actions into hydrogen-bonded chains that are further arranged into distinct layers by weak offset π–π inter­actions.

## Abstract 2

The title compound was synthesized using a novel one-pot method under mild conditions and fully characterized using NMR, ESI–MS and SXRD. The supra­molecular structure of the title compound is defined by a combination of C—H⋯N and π–π inter­actions.

## Chemical context   

The preparation of piperidine derivatives *via* the Mannich reaction is well documented (Noller & Baliah, 1948 ▸). Further, the condensation of a ketone with α-methyl­ene groups, with an aldehyde in the presence of ammonium acetate results in the formation of the required piperidone derivatives through the Mannich reaction (Karthikeyan *et al.*, 2009 ▸; Al-Jeboori *et al.*, 2009 ▸). However, the formation of unpredicted phenanthridine derivatives as a second product with piperidone upon using a range of cyclic ketones has also been mentioned (Karthikeyan *et al.*, 2009 ▸). Phenanthridine derivatives are an important class of heterocyclic nitro­gen-based compounds that form a range of natural products and biologically important mol­ecules (Tumir *et al.*, 2014 ▸). These compounds have found significant applications in different fields, including their potential applications in medicinal chemistry (Stevens *et al.*, 2008 ▸) and in the fabrication of materials (Gerfaud *et al.*, 2009 ▸). Therefore, researchers have been inter­ested in the development of efficient and versatile methods for the synthesis of these materials (Bao *et al.*, 2014 ▸; Xu *et al.*, 2014 ▸). These compounds can be fabricated using a range of synthetic methods, including cyclization, that require harsh conditions and several preparation steps to obtain phenanthridines (Herrera *et al.*, 2006 ▸). In this paper, the formation of a phenanthridine derivative was achieved *via* a one-pot reaction using cyclo­hexa­none and benzaldehyde in an ethano­lic solution of ammonium acetate.
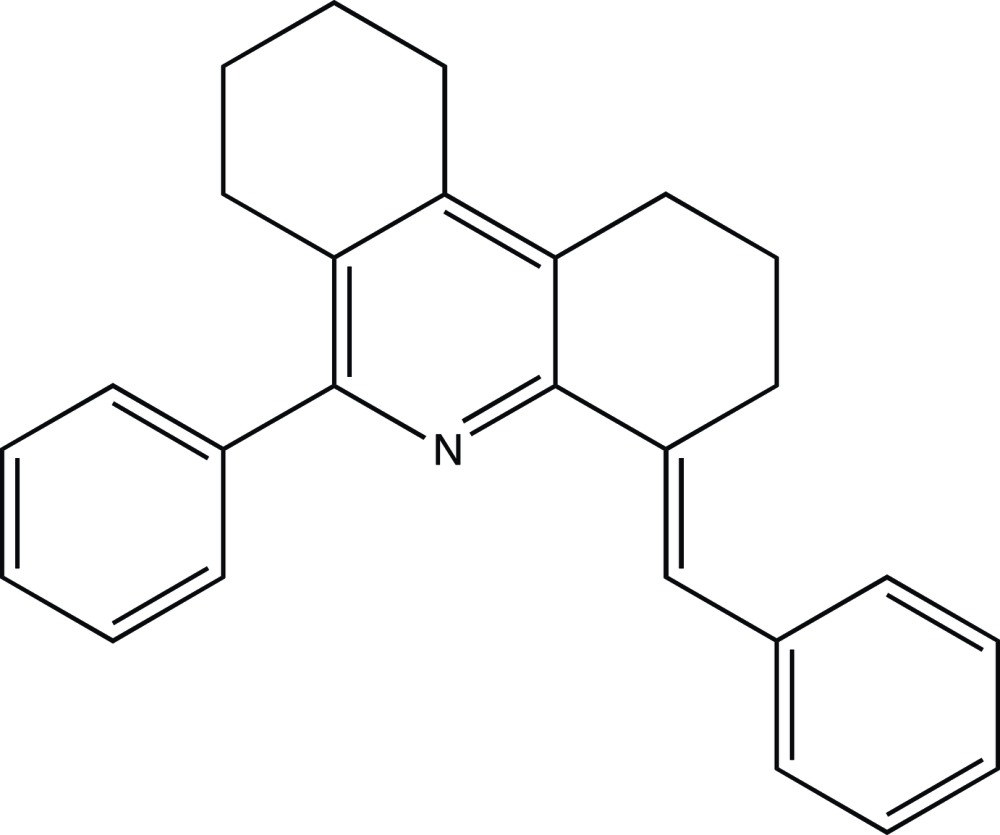


## Structural commentary   

**Figure 1:**
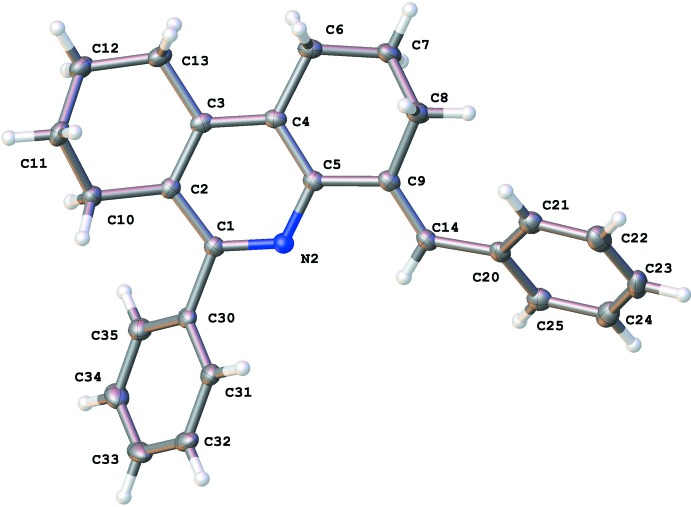
Atom arrangement and numbering scheme for mol­ecule *A*, with displacement ellipsoids drawn at the 50% probability level.

The asymmetric unit contains two crystallographically independent mol­ecules, *A* and *B*, shown in Figs. 1 ▸ and 2 ▸, with no solvent mol­ecules incorporated into the crystal lattice. Selected geometric parameters for the title compound are given in Table 1 ▸. All of the bond lengths and bond angles are within the normal range of analogous phenanthridine compounds (Helesbeux *et al.*, 2011 ▸; Shabashov & Daugulis, 2007 ▸). In the structure, the cyclo­hexane rings adopt the *anti*-envelope conformation. In mol­ecule *B* one of these rings shows static disorder of the C91 and C92 atoms over two sets of sites. This was modelled as two positions with the site occupancies refined to give 81.7 (3)% occupancy for the major component and 18.3 (3)% for the minor component. Full refinement details are given in Section 5. In both of the crystallographically independent mol­ecules, the phenyl and benzyl­idene groups are rotated out-of-plane with respect to the octa­hydro­phenanthrine moieties: in mol­ecule *A* the angle between the mean planes of the phenyl and pyridine rings is 46.92 (5)° with the equivalent angle in mol­ecule *B* of 53.43 (5)°. The angle between the mean planes of the benzyl­idine and pyridine rings in mol­ecule *A* is 48.53 (5)° and the corresponding angle in mol­ecule *B* is 41.37 (5)°.

## Supra­molecular features   

**Figure 3:**
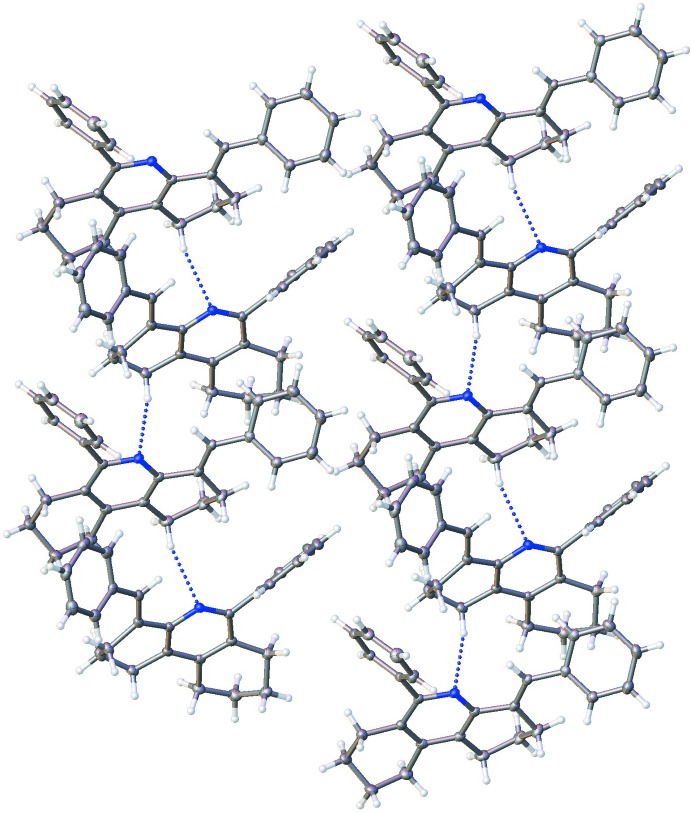
C—H⋯N hydrogen-bonded chains, viewed down the crystallographic *c* axis. The C—H⋯N contacts are shown as dotted blue lines and run along the crystallographic *a* axis.

**Figure 4:**
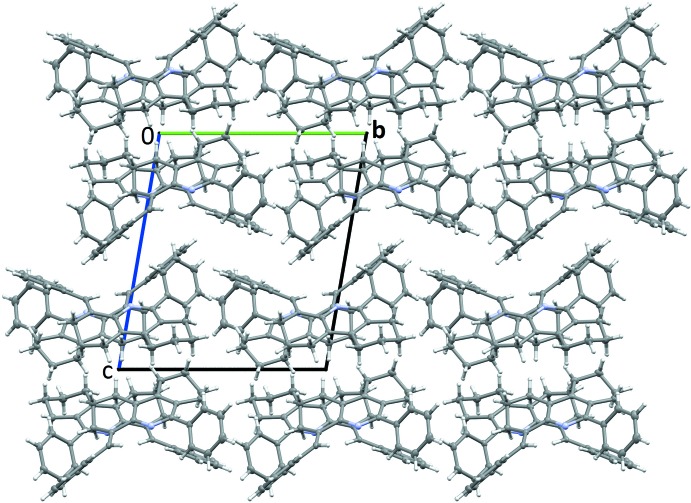
Packing arrangement of the structure viewed along the crystallographic *a* axis with the *c* axis parallel to the long axis of the paper. The π–π inter­actions occur between the benzyl rings that lie between the second and third rows of mol­ecules The labels of the axes should be larger.

The crystal structure features a combination of weak hydrogen bonds and weak offset π–π inter­actions. A weak C—H⋯N contact is formed from the octa­hydro­phenanthridine C6 position in mol­ecule *A* to the N1 position in a *B* mol­ecule (symmetry operation 1 + *x*, −1 + *y*, *z*), with an equivalent weak contact formed from the C109 position in mol­ecule *B* to the N2 position of a neighbouring mol­ecule *A* (symmetry operation 1 – x, 2 − *y*, *z*). Geometric parameters for these contacts are given in Table 2 ▸. The geometric parameters for these contacts are within the accepted range of *D*⋯*A* distances for weak hydrogen bonds of 3.2–4.0 Å, the *D*—H⋯*A* angles being slightly more linear than the expected values of 90–150° (Gilli, 2002 ▸). These inter­actions lead to the formation of chains consisting of alternating *A* and *B* mol­ecules oriented along the *a*-axis direction. These chains propagate along the *b*axis, with neighbouring chains offset from each other along the *a* axis to allow inter­calation of the phenyl and benzyl aromatic rings of neighbouring groups, as shown in Fig 3 ▸, forming layers. These layers further stack along the *c*-axis with the orientation of the layers inverted with respect to the layer above and below, as shown in Fig. 4 ▸. The structure is further stabilized by along the b-axis stabilized by weak offset π–π stacking inter­actions between the benzyl­idine rings of *B* mol­ecules in adjacent layers where the aromatic groups are oriented towards each other (symmetry operation for second *B* mol­ecule 1 − *x*, −*y*, 1 − *z*) with a centroid–centroid distance of 3.9853 (14) Å and shift distance of 2.285 (3) Å.

## Database survey   

Version 5.38 of the Cambridge Structural Database (CSD; Groom *et al.*, 2016 ▸) was queried for inter­molecular C—H⋯N inter­actions between cyclo­hexyl and pyridyl groups with H-atom positions normalized and metals excluded with H⋯N distances restricted to vdW + 0.5 Å. 198 hits were obtained with the minimum and maximum H⋯N contact distances of 2.421 Å and 3.246 Å respectively with a median distance of 2.866 Å and mean of 2.853 Å. The C—H⋯N angles ranged from 92 to 174° with a mean of 128° and a median of 127°. The C—H⋯N contacts for the two crystallographically independent mol­ecules in this work are therefore shorter and more linear than the average, indicating a non-trivial role in determining the supra­molecular structure.

## Hirschfeld surface analysis   

**Figure 5:**
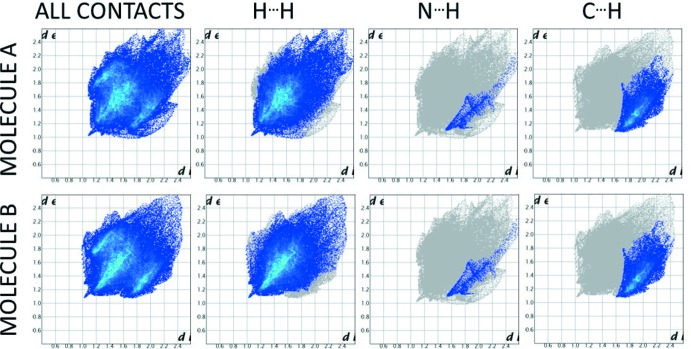
Hirschfeld surface fingerprint plots generated from the *d*_norm_ surfaces generated for mol­ecules *A* and *B* in *CrystalExplorer* at high resolution. The decomposed plots show the areas of contact between H atoms (inter­nal) and H, C and N atoms (external).

Fingerprint analysis of the inter­molecular inter­actions by the generation of Hirschfeld surfaces using *CrystalExplorer* (Spackman & McKinnon, 2002 ▸) reveals that the two types of mol­ecules have similar inter­molecular contact patterns. Selected fingerprint plots corresponding to the complete inter­molecular contact surface and H⋯H, H⋯C and H⋯N contacts are shown in Fig. 5 ▸. The percentage contributions of each contact type to the overall inter­action environment are tabulated in Table 3 ▸. In both cases, the major contribution is from H⋯H contacts, accounting for 66.9% of the surface area in mol­ecule *A* and 64.8% in mol­ecule *B*. It is notable that, in addition to making the largest contribution to the inter­molecular contact surfaces, the H⋯H contacts account for the closest inter­molecular contact in the case of both mol­ecules, between cyclo­hexyl hydrogen atoms on a mol­ecule *A* and *B* (H91*B*⋯H10*X*). The direction of these contacts runs parallel to the axis of the C—H⋯N contacts between mol­ecules on neighbouring hydrogen-bonded chains and appears to result from the inter­calation of these chains. As these contacts are not associated with either of the major attractive inter­actions (*A*–*B* C—H⋯N hydrogen bonds or *B*–*B* π–π stacking), it is probable that this contact arises solely from the packing arrangement required to maximize the number and strength of these favourable inter­actions.

## Synthesis and crystallization   

**Figure 6:**
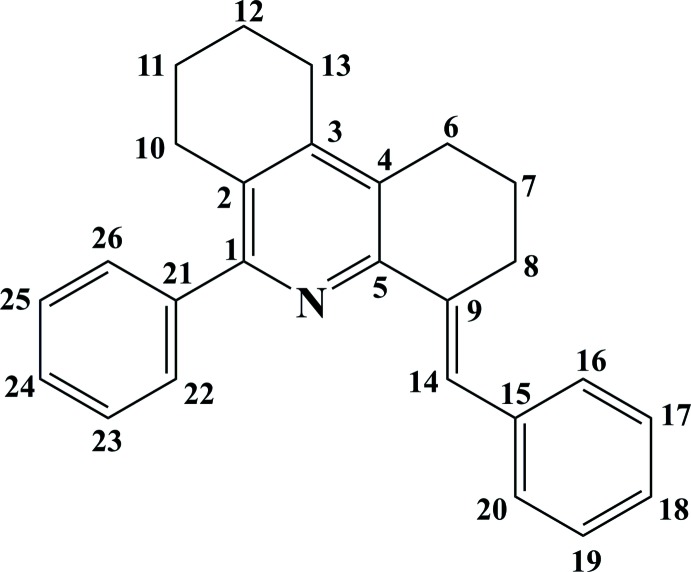
General numbering pattern for NMR spectra of the title compound.

The title compound was isolated from the reaction mixture using a flash column chromatography and as follows: A solution of benzaldehyde (4.02 mL, 0.038 mol), ammonium acetate (1 g, 0.019 mol) and cyclo­hexa­none (2 mL, 0.019 mol) in ethanol (20 mL) was heated to reflux for 2 h. The obtained residue was purified from the crude product by flash chromatography with an eluent mixture of 33% ethyl acetate in hexane, m.p. = 467–469 K, yield: 42%. Colourless crystals suitable for X-ray single crystal analysis were obtained by slow evaporation of a methanol solution of the compound.

(IR, KBr) cm^−1^: 1600 ν (C=N), 1508 ν (C=C)aromatic ring. NMR data (ppm) (numbering scheme shown in Fig. 6 ▸); ^1^H NMR, *δ*_H_ (400 MHz, DMSO-*d*_6_): 7.81 (*s*, 1H, H-14), 7.52–7.35 (*m*, 9H, Ar-H), 7.28–7.21 (*m*, 1H, Ar-H), 2.79 (*t*, 2H, H-13, *J* = 10.4Hz), 2.71 (*t*, 2H, H-6, *J* = 12Hz), 2.66 (*t*, 2H, H-10, *J* = 12.8Hz), 2.61 (*t*, 2H, H-8, *J* = 12.8Hz), 1.80 (*m*, 4H, H-11;12), 1.62 (*m*, 2H, H-7). ^13^C NMR, *δ*_c_ (100 MHz, DMSO-*d*_6_): 155.15 (C-1), 147.72 (C-5), 144.86 (C-3), 140.98 (C-9) and 137.45 (C-2), 136.07 (C-15), 129.61 (C-4), 129.44 (C-21), 129.14, 129.03, 128.76, 128.33, 128.00 and 127.76 and 126.70 (C-Ar), 124.60 (C-14), 27.83 C-8), 26.90 (C-6), 25.80 (C-10), 24.91 (C-13), 22.18 (C-11;12), 21.96 (C-7). The electrospray (+) mass spectrum showed the parent ion peak at *m*/*z* = 352.2068 (*M* + H)^+^ for C_26_H_26_N; requires =352.2065. Elemental analysis: calculated for C_26_H_25_N: C 88.85%, H 7.17%, N 3.99%; found: C 88.76%, H 7.20%, N 3.88%.

## Refinement   

Crystal data, data collection and structure refinement details are summarized in Table 4 ▸. Hydrogen atoms were positioned geometrically (C—H = 0.95–0.99 Å) and refined using a riding model with *U_i_*_so_(H)= 1.2*U*_eq_(C). Disorder at C90/C91/C92/C93 was modelled by splitting the component atoms across two positions and refining the occupancy using FVAR to 82% for C90*A*–C93*A* and 12% for C90*B*–C93*B*. 1,2 distances were restrained using SADI and ADPs for C90*A*/C90*B* and C93*A*/C93*B* constrained using EADP commands.

## Supplementary Material

Crystal structure: contains datablock(s) I. DOI: 10.1107/S2056989017009537/ff2149sup1.cif

Structure factors: contains datablock(s) I. DOI: 10.1107/S2056989017009537/ff2149Isup2.hkl

Supporting information file. DOI: 10.1107/S2056989017009537/ff2149Isup3.cml

CCDC reference: 1506784

Additional supporting information:  crystallographic information; 3D view; checkCIF report

## Figures and Tables

**Figure 2:**
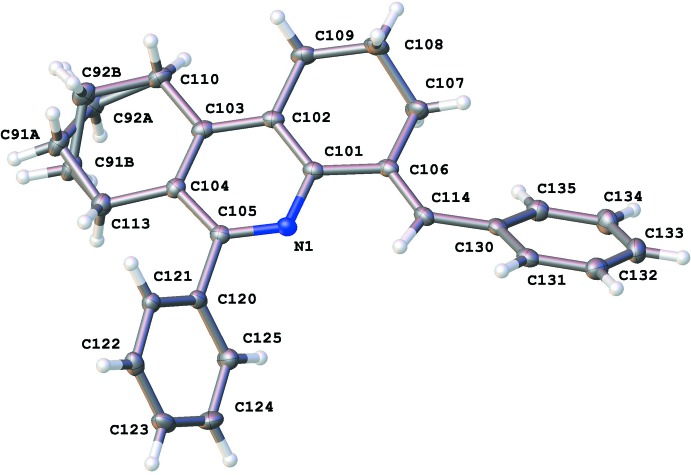
Atom arrangement and numbering scheme for mol­ecule *B*, with displacement ellipsoids drawn at the 50% probability level.

**Table 1:** Selected geometric parameters (Å, °)

C1—N2	1.3351 (19)	C101—N1	1.3492 (18)
C5—N2	1.3511 (18)	C105—N1	1.3308 (19)
C14—C9—C5	120.36 (13)	C106—C114—C130	128.70 (14)
C9—C14—C20	128.41 (14)	C105—N1—C101	119.51 (12)
C114—C106—C101	119.35 (13)	C1—N2—C5	119.35 (12)

**Table 2:** Hydrogen-bond geometry (Å, °)

*D*—H⋯*A*	*D*—H	H⋯*A*	*D*⋯*A*	*D*—H⋯*A*
C6—H6*A*⋯N1^i^	0.97	2.77 (1)	3.672 (2)	155 (1)
C109—H10*B*⋯N2^i^	0.97	2.74 (1)	3.6756 (18)	163 (1)

Symmetry code: (i) 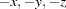.

**Table 3:** Percentage of *d*_norm_ Hirschfeld surface accounted for by each (int)–(ext) contact type

Contact (int)–(ext)	H⋯H	H⋯C	H⋯N	C⋯H	N⋯H
Mol­ecule *A*	66.9%	12.8%	1.3%	16.5%	1.5%
Mol­ecule *B*	64.8%	14.5%	1.3%	17.9%	1.5%

**Table 4:** Experimental details

Crystal data	
Chemical formula	C_26_H_25_N
*M*_r_	351.47
Crystal system, space group	Triclinic, *P* 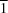
Temperature (K)	100
*a*, *b*, *c* (Å)	11.0758 (8), 12.4989 (11), 14.2425 (13)
α, β, γ (°)	98.088 (3), 96.537 (3), 102.151 (3)
*V* (Å^3^)	1887.2 (3)
*Z*	4
Radiation type	Mo *K*α
μ (mm^−1^)	0.07
Crystal size (mm)	0.73 × 0.12 × 0.10

Data collection	
Diffractometer	Bruker APEX II CCD
Absorption correction	–
No. of measured, independent and observed [*I* > 2σ(*I*)] reflections	37669, 9087, 6437
*R*_int_	0.052
(sin θ/λ)_max_ (Å^−1^)	0.661

Refinement	
*R*[*F* ^2^ > 2σ(*F* ^2^)], *wR*(*F* ^2^), *S*	0.049, 0.131, 1.04
No. of reflections	9087
No. of parameters	500
No. of restraints	7
H-atom treatment	H-atom parameters constrained
Δρ_max_, Δρ_min_ (e Å^−3^)	0.28, −0.36

Computer programs: *APEX2* and *SAINT* (Bruker, 2014 ▸), *SHELXS97* (Sheldrick 2008 ▸), *SHELXL2016* (Sheldrick, 2015 ▸) and *OLEX2* (Dolomanov *et al.*, 2009 ▸).

## supplementary crystallographic information

### Crystal data

**Table:**

C_26_H_25_N	*Z* = 4
*M**_r_* = 351.47	*F*(000) = 752
Triclinic, *P*1	*D*_x_ = 1.237 Mg m^−^^3^
*a* = 11.0758 (8) Å	Mo *K*α radiation, λ = 0.71073 Å
*b* = 12.4989 (11) Å	Cell parameters from 8882 reflections
*c* = 14.2425 (13) Å	θ = 3.7–28.2°
α = 98.088 (3)°	µ = 0.07 mm^−^^1^
β = 96.537 (3)°	*T* = 100 K
γ = 102.151 (3)°	Needle, colourless
*V* = 1887.2 (3) Å^3^	0.73 × 0.12 × 0.10 mm

### Data collection

**Table:**

Bruker APEX II CCD diffractometer	6437 reflections with *I* > 2σ(*I*)
Radiation source: sealed tube	*R*_int_ = 0.052
Graphite monochromator	θ_max_ = 28.0°, θ_min_ = 1.5°
Detector resolution: 8 pixels mm^-1^	*h* = −14→12
ω and φ scans	*k* = −16→16
37669 measured reflections	*l* = −18→18
9087 independent reflections	

### Refinement

**Table:**

Refinement on *F*^2^	7 restraints
Least-squares matrix: full	Hydrogen site location: inferred from neighbouring sites
*R*[*F*^2^ > 2σ(*F*^2^)] = 0.049	H-atom parameters constrained
*wR*(*F*^2^) = 0.131	*w* = 1/[σ^2^(*F*_o_^2^) + (0.0648*P*)^2^ + 0.341*P*] where *P* = (*F*_o_^2^ + 2*F*_c_^2^)/3
*S* = 1.04	(Δ/σ)_max_ < 0.001
9087 reflections	Δρ_max_ = 0.28 e Å^−^^3^
500 parameters	Δρ_min_ = −0.36 e Å^−^^3^

### Special details

**Table:**

Geometry. All esds (except the esd in the dihedral angle between two l.s. planes) are estimated using the full covariance matrix. The cell esds are taken into account individually in the estimation of esds in distances, angles and torsion angles; correlations between esds in cell parameters are only used when they are defined by crystal symmetry. An approximate (isotropic) treatment of cell esds is used for estimating esds involving l.s. planes.
Refinement. Positional disorder at C90-C91-C92-C93 modelled by splitting the component atoms across two positions and refining occupamcy using FVAR to 82% for C90A-C93A and 12% for C90B-C93B. C90A/C90B and C93A/C93B. 1,2 distances were restrained using SADI and ADPs for C90A/C90B and C93A/C93B constrained using EADP commands.

### Fractional atomic coordinates and isotropic or equivalent isotropic displacement parameters (Å^2^)

**Table:**

	*x*	*y*	*z*	*U*_iso_*/*U*_eq_	Occ. (<1)
C1	0.75248 (13)	0.29476 (12)	0.20313 (10)	0.0158 (3)	
C2	0.84704 (13)	0.30527 (12)	0.14419 (10)	0.0159 (3)	
C3	0.88741 (13)	0.20963 (12)	0.11278 (10)	0.0167 (3)	
C4	0.84028 (13)	0.11067 (12)	0.14605 (10)	0.0167 (3)	
C5	0.75826 (13)	0.11251 (12)	0.21406 (10)	0.0162 (3)	
C6	0.88220 (14)	0.00608 (12)	0.11239 (11)	0.0210 (3)	
H6A	0.971308	0.016008	0.138570	0.025*	
H6B	0.874827	−0.005966	0.041569	0.025*	
C7	0.80663 (15)	−0.09584 (13)	0.14287 (11)	0.0228 (3)	
H7A	0.848529	−0.158080	0.131630	0.027*	
H7B	0.722841	−0.118141	0.103657	0.027*	
C8	0.79308 (15)	−0.07234 (13)	0.24859 (11)	0.0226 (3)	
H8A	0.747909	−0.140845	0.268188	0.027*	
H8B	0.876762	−0.048501	0.287910	0.027*	
C9	0.72234 (13)	0.01739 (12)	0.26527 (10)	0.0174 (3)	
C10	0.91203 (13)	0.41798 (13)	0.12489 (11)	0.0190 (3)	
H10G	0.920079	0.474429	0.182854	0.023*	
H10H	0.860508	0.438884	0.072075	0.023*	
C11	1.04157 (14)	0.41736 (13)	0.09788 (11)	0.0215 (3)	
H11A	1.077384	0.488802	0.078186	0.026*	
H11B	1.097725	0.408337	0.153999	0.026*	
C12	1.03130 (15)	0.32257 (13)	0.01614 (11)	0.0237 (4)	
H12A	1.113864	0.326040	−0.005136	0.028*	
H12B	0.972045	0.330066	−0.038744	0.028*	
C13	0.98628 (14)	0.21096 (13)	0.04722 (11)	0.0212 (3)	
H13A	0.952118	0.154118	−0.010738	0.025*	
H13B	1.058869	0.189794	0.080583	0.025*	
C14	0.63212 (13)	0.01532 (12)	0.32117 (11)	0.0182 (3)	
H14	0.589659	0.073877	0.320625	0.022*	
C20	0.59071 (14)	−0.06578 (12)	0.38298 (11)	0.0185 (3)	
C21	0.67174 (14)	−0.11676 (13)	0.43322 (11)	0.0215 (3)	
H21	0.757319	−0.102334	0.425204	0.026*	
C22	0.62901 (16)	−0.18842 (14)	0.49489 (12)	0.0267 (4)	
H22	0.685667	−0.221967	0.528859	0.032*	
C23	0.50500 (16)	−0.21108 (14)	0.50704 (12)	0.0280 (4)	
H23	0.476005	−0.260676	0.548698	0.034*	
C24	0.42291 (15)	−0.16108 (14)	0.45813 (12)	0.0260 (4)	
H24	0.337239	−0.176894	0.465855	0.031*	
C25	0.46554 (14)	−0.08796 (13)	0.39791 (11)	0.0215 (3)	
H25	0.409013	−0.052358	0.366284	0.026*	
C30	0.69251 (13)	0.38769 (12)	0.23336 (11)	0.0173 (3)	
C31	0.67721 (13)	0.41515 (13)	0.32883 (11)	0.0199 (3)	
H31	0.707041	0.375497	0.375212	0.024*	
C32	0.61888 (14)	0.49979 (13)	0.35682 (12)	0.0247 (4)	
H32	0.610150	0.518423	0.422312	0.030*	
C33	0.57346 (14)	0.55707 (14)	0.29010 (13)	0.0283 (4)	
H33	0.533412	0.614915	0.309501	0.034*	
C34	0.58648 (14)	0.52993 (14)	0.19485 (13)	0.0274 (4)	
H34	0.554651	0.568680	0.148555	0.033*	
C35	0.64618 (14)	0.44587 (13)	0.16682 (12)	0.0222 (3)	
H35	0.655403	0.428021	0.101346	0.027*	
C101	0.24367 (12)	1.06093 (12)	0.22013 (10)	0.0159 (3)	
C102	0.31762 (13)	1.01710 (12)	0.15830 (10)	0.0167 (3)	
C103	0.31864 (13)	0.90403 (13)	0.15121 (10)	0.0175 (3)	
C104	0.24125 (13)	0.83688 (12)	0.20087 (10)	0.0169 (3)	
C105	0.16390 (12)	0.88740 (12)	0.25625 (10)	0.0159 (3)	
C106	0.24962 (13)	1.18171 (12)	0.24183 (10)	0.0167 (3)	
C107	0.34880 (15)	1.25554 (13)	0.20045 (11)	0.0236 (4)	
H10A	0.429983	1.269302	0.242502	0.028*	
H10B	0.326897	1.327928	0.197459	0.028*	
C108	0.36013 (15)	1.20115 (13)	0.10034 (11)	0.0240 (4)	
H10C	0.423462	1.251491	0.073296	0.029*	
H10D	0.279151	1.188266	0.058194	0.029*	
C109	0.39768 (14)	1.09112 (13)	0.10293 (11)	0.0223 (3)	
H10E	0.390784	1.051887	0.036440	0.027*	
H10F	0.486078	1.105900	0.132738	0.027*	
C93A	0.4116 (5)	0.8580 (4)	0.0917 (4)	0.0206 (7)	0.817 (3)
H93A	0.375443	0.841374	0.022929	0.025*	0.817 (3)
H93B	0.490199	0.915521	0.099259	0.025*	0.817 (3)
C90B	0.231 (2)	0.707 (3)	0.203 (2)	0.0180 (7)	0.183 (3)
H90A	0.218318	0.688651	0.267133	0.022*	0.183 (3)
H90B	0.163700	0.659781	0.154055	0.022*	0.183 (3)
C114	0.16908 (13)	1.21776 (12)	0.29555 (11)	0.0173 (3)	
H114	0.106753	1.161469	0.311764	0.021*	
C120	0.07724 (13)	0.82602 (12)	0.31378 (11)	0.0160 (3)	
C121	−0.01618 (13)	0.73238 (12)	0.27271 (11)	0.0171 (3)	
H121	−0.023046	0.703379	0.206497	0.021*	
C122	−0.09929 (13)	0.68110 (13)	0.32762 (11)	0.0205 (3)	
H122	−0.163111	0.617750	0.298720	0.025*	
C123	−0.08947 (14)	0.72189 (14)	0.42419 (12)	0.0240 (4)	
H123	−0.145732	0.686091	0.461845	0.029*	
C124	0.00251 (15)	0.81502 (14)	0.46590 (12)	0.0265 (4)	
H124	0.009518	0.843098	0.532324	0.032*	
C125	0.08462 (14)	0.86754 (13)	0.41083 (11)	0.0220 (3)	
H125	0.146412	0.932388	0.439606	0.026*	
C130	0.16464 (13)	1.33237 (12)	0.33245 (10)	0.0171 (3)	
C131	0.04885 (14)	1.35477 (13)	0.34805 (11)	0.0194 (3)	
H131	−0.023826	1.295852	0.334857	0.023*	
C132	0.03890 (15)	1.46137 (13)	0.38235 (11)	0.0230 (3)	
H132	−0.040504	1.475080	0.391411	0.028*	
C133	0.14399 (15)	1.54818 (14)	0.40355 (12)	0.0261 (4)	
H133	0.136814	1.621421	0.426329	0.031*	
C134	0.25990 (15)	1.52720 (13)	0.39122 (12)	0.0255 (4)	
H134	0.332497	1.586158	0.406367	0.031*	
C135	0.27010 (14)	1.42036 (13)	0.35684 (11)	0.0209 (3)	
H135	0.350105	1.406835	0.349775	0.025*	
N1	0.16703 (11)	0.99549 (10)	0.26679 (9)	0.0163 (3)	
N2	0.71246 (11)	0.20276 (10)	0.23903 (9)	0.0172 (3)	
C91A	0.31965 (18)	0.67122 (17)	0.12626 (16)	0.0232 (5)	0.817 (3)
H91A	0.338416	0.600767	0.140796	0.028*	0.817 (3)
H91B	0.265362	0.655261	0.063378	0.028*	0.817 (3)
C92A	0.44037 (19)	0.75409 (17)	0.12225 (16)	0.0240 (5)	0.817 (3)
H92A	0.492391	0.773150	0.186207	0.029*	0.817 (3)
H92B	0.487991	0.720499	0.076150	0.029*	0.817 (3)
C91B	0.3619 (9)	0.6965 (8)	0.1797 (7)	0.0232 (5)	0.183 (3)
H91C	0.427655	0.745027	0.229720	0.028*	0.183 (3)
H91D	0.369834	0.618866	0.178379	0.028*	0.183 (3)
C92B	0.3780 (10)	0.7300 (8)	0.0831 (7)	0.0240 (5)	0.183 (3)
H92C	0.303890	0.692931	0.034995	0.029*	0.183 (3)
H92D	0.452722	0.709534	0.060388	0.029*	0.183 (3)
C90A	0.2523 (4)	0.7201 (5)	0.2044 (4)	0.0180 (7)	0.817 (3)
H90C	0.167352	0.672261	0.198610	0.022*	0.817 (3)
H90D	0.297639	0.717820	0.267889	0.022*	0.817 (3)
C93B	0.394 (3)	0.8628 (19)	0.100 (2)	0.0206 (7)	0.183 (3)
H93C	0.481093	0.897458	0.130508	0.025*	0.183 (3)
H93D	0.383960	0.885593	0.035915	0.025*	0.183 (3)

### Atomic displacement parameters (Å^2^)

**Table:**

	*U*^11^	*U*^22^	*U*^33^	*U*^12^	*U*^13^	*U*^23^
C1	0.0151 (7)	0.0161 (7)	0.0154 (7)	0.0035 (6)	0.0000 (6)	0.0017 (6)
C2	0.0146 (7)	0.0183 (8)	0.0143 (7)	0.0033 (6)	0.0003 (5)	0.0032 (6)
C3	0.0144 (7)	0.0228 (8)	0.0124 (7)	0.0053 (6)	−0.0002 (5)	0.0018 (6)
C4	0.0147 (7)	0.0185 (8)	0.0162 (7)	0.0053 (6)	0.0003 (6)	−0.0005 (6)
C5	0.0143 (7)	0.0162 (7)	0.0171 (7)	0.0038 (6)	−0.0002 (6)	0.0011 (6)
C6	0.0213 (7)	0.0223 (8)	0.0211 (8)	0.0091 (6)	0.0050 (6)	0.0013 (7)
C7	0.0304 (8)	0.0190 (8)	0.0218 (8)	0.0114 (7)	0.0056 (7)	0.0025 (7)
C8	0.0291 (8)	0.0211 (8)	0.0213 (8)	0.0119 (7)	0.0058 (7)	0.0049 (7)
C9	0.0194 (7)	0.0164 (8)	0.0157 (7)	0.0056 (6)	−0.0009 (6)	0.0012 (6)
C10	0.0212 (7)	0.0192 (8)	0.0173 (8)	0.0043 (6)	0.0048 (6)	0.0043 (6)
C11	0.0205 (7)	0.0246 (8)	0.0199 (8)	0.0023 (6)	0.0055 (6)	0.0076 (7)
C12	0.0249 (8)	0.0291 (9)	0.0210 (8)	0.0090 (7)	0.0092 (7)	0.0080 (7)
C13	0.0213 (7)	0.0262 (9)	0.0188 (8)	0.0088 (7)	0.0070 (6)	0.0048 (7)
C14	0.0197 (7)	0.0143 (7)	0.0198 (8)	0.0045 (6)	−0.0002 (6)	0.0016 (6)
C20	0.0232 (7)	0.0147 (7)	0.0155 (7)	0.0029 (6)	0.0022 (6)	−0.0012 (6)
C21	0.0247 (8)	0.0209 (8)	0.0177 (8)	0.0050 (6)	0.0022 (6)	0.0009 (6)
C22	0.0369 (9)	0.0236 (9)	0.0201 (8)	0.0086 (7)	0.0019 (7)	0.0044 (7)
C23	0.0403 (10)	0.0200 (8)	0.0214 (9)	−0.0005 (7)	0.0077 (7)	0.0050 (7)
C24	0.0252 (8)	0.0242 (9)	0.0233 (9)	−0.0035 (7)	0.0055 (7)	−0.0011 (7)
C25	0.0221 (7)	0.0216 (8)	0.0183 (8)	0.0039 (6)	−0.0001 (6)	−0.0013 (6)
C30	0.0127 (6)	0.0157 (7)	0.0232 (8)	0.0015 (6)	0.0042 (6)	0.0034 (6)
C31	0.0167 (7)	0.0185 (8)	0.0248 (8)	0.0022 (6)	0.0067 (6)	0.0042 (7)
C32	0.0197 (7)	0.0229 (9)	0.0301 (9)	0.0014 (7)	0.0113 (7)	−0.0014 (7)
C33	0.0206 (8)	0.0213 (8)	0.0455 (11)	0.0079 (7)	0.0125 (7)	0.0032 (8)
C34	0.0233 (8)	0.0251 (9)	0.0383 (10)	0.0112 (7)	0.0067 (7)	0.0107 (8)
C35	0.0196 (7)	0.0236 (8)	0.0256 (9)	0.0073 (6)	0.0054 (6)	0.0059 (7)
C101	0.0139 (7)	0.0186 (8)	0.0143 (7)	0.0014 (6)	0.0014 (5)	0.0039 (6)
C102	0.0141 (7)	0.0219 (8)	0.0134 (7)	0.0016 (6)	0.0018 (5)	0.0046 (6)
C103	0.0150 (7)	0.0233 (8)	0.0136 (7)	0.0044 (6)	0.0015 (6)	0.0022 (6)
C104	0.0161 (7)	0.0181 (8)	0.0153 (7)	0.0037 (6)	0.0005 (6)	0.0011 (6)
C105	0.0142 (7)	0.0176 (8)	0.0145 (7)	0.0022 (6)	0.0002 (5)	0.0024 (6)
C106	0.0169 (7)	0.0180 (8)	0.0129 (7)	−0.0005 (6)	0.0001 (6)	0.0032 (6)
C107	0.0259 (8)	0.0209 (8)	0.0221 (8)	−0.0022 (7)	0.0095 (7)	0.0037 (7)
C108	0.0259 (8)	0.0241 (9)	0.0215 (8)	−0.0011 (7)	0.0095 (7)	0.0076 (7)
C109	0.0185 (7)	0.0286 (9)	0.0200 (8)	0.0026 (7)	0.0076 (6)	0.0052 (7)
C93A	0.019 (2)	0.0278 (11)	0.0188 (14)	0.0098 (9)	0.0118 (10)	0.0021 (9)
C90B	0.0137 (19)	0.016 (2)	0.0228 (8)	0.0004 (15)	0.0038 (12)	0.0014 (10)
C114	0.0178 (7)	0.0167 (8)	0.0167 (7)	0.0010 (6)	0.0012 (6)	0.0057 (6)
C120	0.0157 (7)	0.0162 (7)	0.0190 (8)	0.0066 (6)	0.0053 (6)	0.0062 (6)
C121	0.0172 (7)	0.0166 (7)	0.0189 (8)	0.0058 (6)	0.0040 (6)	0.0033 (6)
C122	0.0161 (7)	0.0168 (8)	0.0300 (9)	0.0040 (6)	0.0064 (6)	0.0063 (7)
C123	0.0237 (8)	0.0263 (9)	0.0272 (9)	0.0080 (7)	0.0125 (7)	0.0119 (7)
C124	0.0344 (9)	0.0306 (9)	0.0162 (8)	0.0086 (8)	0.0087 (7)	0.0047 (7)
C125	0.0242 (8)	0.0191 (8)	0.0204 (8)	0.0008 (6)	0.0043 (6)	0.0018 (6)
C130	0.0210 (7)	0.0173 (8)	0.0130 (7)	0.0035 (6)	0.0016 (6)	0.0054 (6)
C131	0.0201 (7)	0.0216 (8)	0.0161 (8)	0.0023 (6)	0.0020 (6)	0.0065 (6)
C132	0.0248 (8)	0.0259 (9)	0.0214 (8)	0.0107 (7)	0.0044 (6)	0.0063 (7)
C133	0.0340 (9)	0.0191 (8)	0.0264 (9)	0.0090 (7)	0.0044 (7)	0.0042 (7)
C134	0.0268 (8)	0.0197 (8)	0.0271 (9)	0.0006 (7)	0.0020 (7)	0.0029 (7)
C135	0.0197 (7)	0.0222 (8)	0.0206 (8)	0.0036 (6)	0.0033 (6)	0.0046 (7)
N1	0.0146 (6)	0.0171 (6)	0.0168 (6)	0.0018 (5)	0.0034 (5)	0.0037 (5)
N2	0.0153 (6)	0.0178 (6)	0.0191 (7)	0.0051 (5)	0.0023 (5)	0.0037 (5)
C91A	0.0211 (10)	0.0225 (10)	0.0253 (12)	0.0076 (8)	0.0045 (9)	−0.0032 (9)
C92A	0.0185 (10)	0.0279 (11)	0.0280 (12)	0.0094 (9)	0.0077 (9)	0.0030 (9)
C91B	0.0211 (10)	0.0225 (10)	0.0253 (12)	0.0076 (8)	0.0045 (9)	−0.0032 (9)
C92B	0.0185 (10)	0.0279 (11)	0.0280 (12)	0.0094 (9)	0.0077 (9)	0.0030 (9)
C90A	0.0137 (19)	0.016 (2)	0.0228 (8)	0.0004 (15)	0.0038 (12)	0.0014 (10)
C93B	0.019 (2)	0.0278 (11)	0.0188 (14)	0.0098 (9)	0.0118 (10)	0.0021 (9)

### Geometric parameters (Å, º)

**Table:**

C1—C2	1.4115 (19)	C23—C24	1.386 (2)
C1—C30	1.492 (2)	C24—H24	0.9500
C93A—H93A	0.9900	C24—C25	1.387 (2)
C93A—H93B	0.9900	C25—H25	0.9500
C90B—H90A	0.9900	C30—C31	1.393 (2)
C90B—H90B	0.9900	C30—C35	1.389 (2)
C1—N2	1.3351 (19)	C31—H31	0.9500
C91A—H91A	0.9900	C31—C32	1.387 (2)
C91A—H91B	0.9900	C32—H32	0.9500
C93A—C92A	1.510 (4)	C32—C33	1.380 (2)
C91A—C92A	1.521 (3)	C33—H33	0.9500
C92A—H92A	0.9900	C33—C34	1.383 (2)
C92A—H92B	0.9900	C34—H34	0.9500
C90B—C91B	1.55 (2)	C34—C35	1.391 (2)
C91B—H91C	0.9900	C35—H35	0.9500
C91B—H91D	0.9900	C101—C102	1.4014 (19)
C91B—C92B	1.513 (11)	C101—C106	1.484 (2)
C92B—H92C	0.9900	C101—N1	1.3492 (18)
C92B—H92D	0.9900	C102—C103	1.405 (2)
C91A—C90A	1.536 (5)	C102—C109	1.511 (2)
C90A—H90C	0.9900	C103—C104	1.393 (2)
C90A—H90D	0.9900	C103—C93A	1.556 (4)
C92B—C93B	1.61 (2)	C103—C93B	1.31 (2)
C93B—H93C	0.9900	C104—C105	1.4128 (19)
C93B—H93D	0.9900	C104—C90B	1.61 (4)
C2—C3	1.396 (2)	C104—C90A	1.498 (7)
C2—C10	1.518 (2)	C105—C120	1.492 (2)
C3—C4	1.406 (2)	C105—N1	1.3308 (19)
C3—C13	1.5168 (19)	C106—C107	1.510 (2)
C4—C5	1.4025 (19)	C106—C114	1.3460 (19)
C4—C6	1.511 (2)	C107—H10A	0.9900
C5—C9	1.488 (2)	C107—H10B	0.9900
C5—N2	1.3511 (18)	C107—C108	1.521 (2)
C6—H6A	0.9900	C108—H10C	0.9900
C6—H6B	0.9900	C108—H10D	0.9900
C6—C7	1.517 (2)	C108—C109	1.522 (2)
C7—H7A	0.9900	C109—H10E	0.9900
C7—H7B	0.9900	C109—H10F	0.9900
C7—C8	1.524 (2)	C114—H114	0.9500
C8—H8A	0.9900	C114—C130	1.468 (2)
C8—H8B	0.9900	C120—C121	1.394 (2)
C8—C9	1.505 (2)	C120—C125	1.393 (2)
C9—C14	1.345 (2)	C121—H121	0.9500
C10—H10G	0.9900	C121—C122	1.388 (2)
C10—H10H	0.9900	C122—H122	0.9500
C10—C11	1.5285 (19)	C122—C123	1.381 (2)
C11—H11A	0.9900	C123—H123	0.9500
C11—H11B	0.9900	C123—C124	1.384 (2)
C11—C12	1.516 (2)	C124—H124	0.9500
C12—H12A	0.9900	C124—C125	1.389 (2)
C12—H12B	0.9900	C125—H125	0.9500
C12—C13	1.524 (2)	C130—C131	1.4040 (19)
C13—H13A	0.9900	C130—C135	1.398 (2)
C13—H13B	0.9900	C131—H131	0.9500
C14—H14	0.9500	C131—C132	1.385 (2)
C14—C20	1.470 (2)	C132—H132	0.9500
C20—C21	1.395 (2)	C132—C133	1.385 (2)
C20—C25	1.402 (2)	C133—H133	0.9500
C21—H21	0.9500	C133—C134	1.388 (2)
C21—C22	1.391 (2)	C134—H134	0.9500
C22—H22	0.9500	C134—C135	1.388 (2)
C22—C23	1.379 (2)	C135—H135	0.9500
C23—H23	0.9500		
			
C2—C1—C30	122.39 (13)	C24—C23—H23	120.2
N2—C1—C2	123.03 (13)	C23—C24—H24	119.9
N2—C1—C30	114.54 (12)	C23—C24—C25	120.16 (15)
C1—C2—C10	121.26 (12)	C25—C24—H24	119.9
C3—C2—C1	117.21 (13)	C20—C25—H25	119.5
C3—C2—C10	121.18 (12)	C24—C25—C20	121.07 (15)
C2—C3—C4	119.60 (12)	C24—C25—H25	119.5
C92A—C93A—C103	112.2 (2)	C31—C30—C1	120.48 (13)
C91B—C90B—C104	100.5 (13)	C35—C30—C1	121.05 (13)
C92A—C93A—H93A	109.2	C35—C30—C31	118.43 (13)
C92A—C93A—H93B	109.2	C30—C31—H31	119.7
H93A—C93A—H93B	107.9	C32—C31—C30	120.65 (15)
C92B—C91B—C90B	109.1 (13)	C32—C31—H31	119.7
C91B—C90B—H90A	111.7	C31—C32—H32	119.8
C91B—C90B—H90B	111.7	C33—C32—C31	120.37 (15)
C2—C3—C13	121.57 (13)	C33—C32—H32	119.8
C4—C3—C13	118.76 (13)	C32—C33—H33	120.1
C3—C4—C6	120.39 (12)	C32—C33—C34	119.71 (14)
C5—C4—C3	118.62 (13)	C34—C33—H33	120.1
C5—C4—C6	120.93 (13)	C33—C34—H34	120.0
C4—C5—C9	121.62 (13)	C33—C34—C35	120.00 (16)
N2—C5—C4	121.40 (13)	C35—C34—H34	120.0
N2—C5—C9	116.92 (12)	C30—C35—C34	120.84 (15)
C4—C6—H6A	109.0	C30—C35—H35	119.6
C4—C6—H6B	109.0	C34—C35—H35	119.6
C4—C6—C7	112.79 (12)	C102—C101—C106	122.24 (13)
H6A—C6—H6B	107.8	C103—C93A—H93A	109.2
C7—C6—H6A	109.0	N1—C101—C102	121.45 (13)
C7—C6—H6B	109.0	N1—C101—C106	116.24 (12)
C6—C7—H7A	109.5	C101—C102—C103	118.68 (13)
C6—C7—H7B	109.5	C101—C102—C109	120.45 (13)
C6—C7—C8	110.76 (13)	C103—C102—C109	120.85 (13)
H90A—C90B—H90B	109.4	C102—C103—C93A	118.83 (18)
C93A—C92A—C91A	110.0 (2)	C104—C103—C102	119.70 (13)
C92A—C91A—H91A	109.8	C104—C103—C93A	121.41 (18)
C90A—C91A—H91A	109.8	C103—C104—C105	117.24 (13)
H91A—C91A—H91B	108.3	C103—C104—C90B	126.8 (6)
C92A—C91A—H91B	109.8	C103—C104—C90A	120.55 (16)
C90A—C91A—H91B	109.8	C105—C104—C90B	115.9 (6)
C91A—C92A—H92A	109.7	C105—C104—C90A	121.79 (17)
C93A—C92A—H92A	109.7	C104—C105—C120	123.11 (13)
C93A—C92A—H92B	109.7	N1—C105—C104	123.19 (13)
C91A—C92A—H92B	109.7	N1—C105—C120	113.58 (12)
H92A—C92A—H92B	108.2	C101—C106—C107	115.73 (12)
C90B—C91B—H91C	109.9	C114—C106—C101	119.35 (13)
C92B—C91B—H91C	109.9	C114—C106—C107	124.93 (14)
C92B—C91B—H91D	109.9	C106—C107—H10A	109.6
H91C—C91B—H91D	108.3	C106—C107—H10B	109.6
C90B—C91B—H91D	109.9	C106—C107—C108	110.36 (13)
C91B—C92B—H92C	110.6	H10A—C107—H10B	108.1
C93B—C92B—H92C	110.6	C108—C107—H10A	109.6
C93B—C92B—H92D	110.6	C108—C107—H10B	109.6
H92C—C92B—H92D	108.8	C103—C93A—H93B	109.2
H7A—C7—H7B	108.1	C103—C93B—C92B	117.3 (13)
C8—C7—H7A	109.5	C103—C93B—H93C	108.0
C8—C7—H7B	109.5	C103—C93B—H93D	108.0
C7—C8—H8A	109.7	C104—C90B—H90A	111.7
C7—C8—H8B	109.7	C104—C90B—H90B	111.7
H8A—C8—H8B	108.2	C104—C90A—C91A	114.6 (3)
C9—C8—C7	109.87 (13)	C104—C90A—H90C	108.6
C9—C8—H8A	109.7	C104—C90A—H90D	108.6
C9—C8—H8B	109.7	C107—C108—H10C	109.5
C5—C9—C8	115.10 (12)	C107—C108—H10D	109.5
C14—C9—C5	120.36 (13)	C107—C108—C109	110.87 (13)
C14—C9—C8	124.55 (14)	H10C—C108—H10D	108.1
C2—C10—H10G	109.3	C109—C108—H10C	109.5
C2—C10—H10H	109.3	C109—C108—H10D	109.5
C2—C10—C11	111.63 (12)	C102—C109—C108	112.51 (12)
H10G—C10—H10H	108.0	C102—C109—H10E	109.1
C11—C10—H10G	109.3	C102—C109—H10F	109.1
C11—C10—H10H	109.3	C108—C109—H10E	109.1
C10—C11—H11A	109.8	C108—C109—H10F	109.1
C10—C11—H11B	109.8	H10E—C109—H10F	107.8
H11A—C11—H11B	108.2	C106—C114—H114	115.7
C12—C11—C10	109.45 (13)	C106—C114—C130	128.70 (14)
C12—C11—H11A	109.8	C130—C114—H114	115.7
C12—C11—H11B	109.8	C121—C120—C105	122.34 (13)
C11—C12—H12A	109.5	C125—C120—C105	118.96 (13)
C11—C12—H12B	109.5	C125—C120—C121	118.57 (13)
C11—C12—C13	110.83 (12)	C120—C121—H121	119.7
H12A—C12—H12B	108.1	C122—C121—C120	120.64 (14)
C13—C12—H12A	109.5	C122—C121—H121	119.7
C13—C12—H12B	109.5	C121—C122—H122	119.9
C3—C13—C12	114.32 (12)	C123—C122—C121	120.20 (15)
C3—C13—H13A	108.7	C123—C122—H122	119.9
C3—C13—H13B	108.7	C122—C123—H123	120.1
C12—C13—H13A	108.7	C122—C123—C124	119.83 (14)
C12—C13—H13B	108.7	C124—C123—H123	120.1
H13A—C13—H13B	107.6	C123—C124—H124	119.9
C9—C14—H14	115.8	C123—C124—C125	120.11 (15)
C9—C14—C20	128.41 (14)	C125—C124—H124	119.9
C20—C14—H14	115.8	C120—C125—H125	119.7
C21—C20—C14	123.19 (13)	C124—C125—C120	120.63 (15)
C21—C20—C25	117.75 (14)	C124—C125—H125	119.7
C25—C20—C14	118.91 (14)	C131—C130—C114	118.49 (13)
C20—C21—H21	119.5	C135—C130—C114	123.77 (13)
C22—C21—C20	120.94 (14)	C135—C130—C131	117.69 (14)
C22—C21—H21	119.5	C130—C131—H131	119.5
C21—C22—H22	119.8	C132—C131—C130	121.04 (14)
C23—C22—C21	120.45 (16)	C132—C131—H131	119.5
C23—C22—H22	119.8	C131—C132—H132	119.8
C22—C23—H23	120.2	C131—C132—C133	120.43 (14)
C91B—C92B—H92D	110.6	C133—C132—H132	119.8
C92A—C91A—C90A	109.2 (2)	C132—C133—H133	120.3
C91A—C90A—H90C	108.6	C132—C133—C134	119.42 (15)
C91A—C90A—H90D	108.6	C134—C133—H133	120.3
H90C—C90A—H90D	107.6	C133—C134—H134	119.9
C91B—C92B—C93B	105.6 (12)	C135—C134—C133	120.25 (15)
C92B—C93B—H93C	108.0	C135—C134—H134	119.9
C92B—C93B—H93D	108.0	C130—C135—H135	119.4
H93C—C93B—H93D	107.2	C134—C135—C130	121.11 (14)
C93B—C103—C102	119.9 (8)	C134—C135—H135	119.4
C93B—C103—C104	120.4 (8)	C105—N1—C101	119.51 (12)
C22—C23—C24	119.60 (15)	C1—N2—C5	119.35 (12)
			
C90B—C91B—C92B—C93B	71.8 (19)	C32—C33—C34—C35	−0.6 (2)
C90A—C91A—C92A—C93A	−64.4 (4)	C33—C34—C35—C30	0.5 (2)
C91B—C92B—C93B—C103	−44 (3)	C35—C30—C31—C32	−1.0 (2)
C92A—C91A—C90A—C104	48.1 (4)	C101—C102—C103—C104	3.8 (2)
C1—C2—C3—C4	−4.7 (2)	C101—C102—C109—C108	−16.2 (2)
C1—C2—C3—C13	178.50 (13)	C101—C106—C107—C108	38.53 (18)
C1—C2—C10—C11	155.77 (14)	C101—C106—C114—C130	174.32 (14)
C1—C30—C31—C32	−178.48 (13)	C102—C101—C106—C107	−5.4 (2)
C1—C30—C35—C34	177.76 (14)	C102—C101—C106—C114	174.64 (14)
C93B—C103—C104—C90B	0 (2)	C102—C101—N1—C105	2.0 (2)
C101—C102—C103—C93B	−174.8 (18)	C102—C103—C93A—C92A	156.5 (3)
C101—C102—C103—C93A	−173.2 (3)	C102—C103—C93B—C92B	−172.1 (13)
C93A—C103—C104—C90A	4.7 (4)	C102—C103—C104—C105	0.4 (2)
C109—C102—C103—C93B	3.3 (18)	C103—C93A—C92A—C91A	49.9 (5)
C109—C102—C103—C93A	4.8 (3)	C103—C102—C109—C108	165.74 (14)
C93A—C103—C104—C105	177.3 (3)	C103—C104—C90B—C91B	25 (2)
C102—C103—C104—C90B	−178.8 (14)	C103—C104—C90A—C91A	−18.9 (5)
C102—C103—C104—C90A	−172.3 (3)	C103—C104—C105—C120	−179.43 (13)
C93B—C103—C104—C105	178.9 (18)	C90B—C104—C105—N1	175.5 (13)
C2—C1—C30—C31	−132.86 (15)	C90A—C104—C105—N1	168.8 (3)
C2—C1—C30—C35	49.7 (2)	C103—C104—C105—N1	−3.7 (2)
C2—C1—N2—C5	−4.0 (2)	C104—C90B—C91B—C92B	−59.3 (17)
C2—C3—C4—C5	−3.2 (2)	C104—C103—C93A—C92A	−20.5 (6)
C2—C3—C4—C6	179.61 (13)	C104—C103—C93B—C92B	9 (3)
C2—C3—C13—C12	−2.7 (2)	C104—C105—C120—C121	−57.7 (2)
C2—C10—C11—C12	52.66 (17)	C104—C105—C120—C125	126.44 (16)
C3—C2—C10—C11	−17.37 (19)	C104—C105—N1—C101	2.6 (2)
C3—C4—C5—C9	−169.02 (13)	C105—C104—C90B—C91B	−154.5 (9)
C3—C4—C5—N2	8.1 (2)	C105—C104—C90A—C91A	168.8 (2)
C3—C4—C6—C7	−170.74 (13)	C105—C120—C121—C122	−176.48 (13)
C4—C3—C13—C12	−179.58 (13)	C105—C120—C125—C124	177.61 (14)
C4—C5—C9—C8	7.9 (2)	C106—C101—C102—C103	171.76 (13)
C4—C5—C9—C14	−171.80 (14)	C106—C101—C102—C109	−6.3 (2)
C4—C5—N2—C1	−4.6 (2)	C106—C101—N1—C105	−175.10 (13)
C4—C6—C7—C8	−47.05 (17)	C106—C107—C108—C109	−60.97 (17)
C5—C4—C6—C7	12.1 (2)	C106—C114—C130—C131	151.65 (16)
C5—C9—C14—C20	−174.65 (14)	C106—C114—C130—C135	−31.1 (2)
C6—C4—C5—C9	8.2 (2)	C107—C106—C114—C130	−5.6 (3)
C6—C4—C5—N2	−174.71 (13)	C107—C108—C109—C102	49.52 (18)
C6—C7—C8—C9	62.67 (17)	C109—C102—C103—C104	−178.16 (14)
C7—C8—C9—C5	−42.45 (18)	C114—C106—C107—C108	−141.57 (15)
C7—C8—C9—C14	137.19 (16)	C114—C130—C131—C132	−179.71 (14)
C8—C9—C14—C20	5.7 (3)	C114—C130—C135—C134	179.84 (14)
C9—C5—N2—C1	172.70 (13)	C120—C105—N1—C101	178.66 (12)
C9—C14—C20—C21	35.3 (2)	C120—C121—C122—C123	−0.6 (2)
C9—C14—C20—C25	−149.20 (16)	C121—C120—C125—C124	1.6 (2)
C10—C2—C3—C4	168.74 (13)	C121—C122—C123—C124	0.8 (2)
C90A—C104—C105—C120	−6.9 (3)	C122—C123—C124—C125	0.1 (2)
C90B—C104—C105—C120	−0.2 (13)	C123—C124—C125—C120	−1.4 (2)
C10—C2—C3—C13	−8.1 (2)	C125—C120—C121—C122	−0.6 (2)
C10—C11—C12—C13	−64.02 (16)	C130—C131—C132—C133	−1.1 (2)
C11—C12—C13—C3	38.47 (18)	C131—C130—C135—C134	−2.8 (2)
C13—C3—C4—C5	173.73 (13)	C131—C132—C133—C134	−0.8 (2)
C13—C3—C4—C6	−3.5 (2)	C132—C133—C134—C135	0.8 (2)
C14—C20—C21—C22	176.47 (14)	C133—C134—C135—C130	1.1 (2)
C14—C20—C25—C24	−177.86 (14)	C135—C130—C131—C132	2.8 (2)
C20—C21—C22—C23	0.5 (2)	N1—C101—C102—C103	−5.1 (2)
C21—C20—C25—C24	−2.1 (2)	N1—C101—C102—C109	176.83 (13)
C21—C22—C23—C24	−0.7 (2)	N1—C101—C106—C107	171.57 (13)
C22—C23—C24—C25	−0.5 (2)	N1—C101—C106—C114	−8.3 (2)
C23—C24—C25—C20	2.0 (2)	N1—C105—C120—C121	126.25 (15)
C25—C20—C21—C22	0.9 (2)	N1—C105—C120—C125	−49.63 (18)
C30—C1—C2—C3	−173.79 (13)	N2—C1—C2—C3	8.6 (2)
C30—C1—C2—C10	12.8 (2)	N2—C1—C2—C10	−164.80 (14)
C30—C1—N2—C5	178.20 (13)	N2—C1—C30—C31	44.94 (19)
C30—C31—C32—C33	0.9 (2)	N2—C1—C30—C35	−132.46 (15)
C31—C30—C35—C34	0.3 (2)	N2—C5—C9—C8	−169.40 (13)
C31—C32—C33—C34	−0.1 (2)	N2—C5—C9—C14	11.0 (2)

### Hydrogen-bond geometry (Å, º)

**Table:**

*D*—H···*A*	*D*—H	H···*A*	*D*···*A*	*D*—H···*A*
C6—H6*A*···N1^i^	0.97	2.77 (1)	3.672 (2)	155 (1)
C109—H10*B*···N2^i^	0.97	2.74 (1)	3.6756 (18)	163 (1)

Symmetry code: (i) −*x*, −*y*, −*z*.
